# Impact of Chronic Hepatitis C Virus Genotype 1b Infection on Triglyceride Concentration in Serum Lipoprotein Fractions

**DOI:** 10.3390/ijms160920576

**Published:** 2015-08-31

**Authors:** Tomohisa Nagano, Nobuyoshi Seki, Yoichi Tomita, Tomonori Sugita, Yuta Aida, Munenori Itagaki, Satoshi Sutoh, Hiroshi Abe, Akihito Tsubota, Yoshio Aizawa

**Affiliations:** 1 Department of Gastroenterology and Hepatology Internal Medicine, Jikei University Katsushika Medical Center, 6-41-2 Aoto, Katsushika-ku, Tokyo 125-8506, Japan; E-Mails: novseki@jikei.ac.jp (N.S.); youichi_0618@yahoo.co.jp (Y.T.); tonexpress@hotmail.com (T.S.); yutaaida@gmail.com (Y.A.); munenori1004@jikei.ac.jp (M.I.); sutoh@jikei.ac.jp (S.S.); hiroshiabe222@aol.com (H.A.); aichanyoshi@yahoo.co.jp (Y.A.); 2 Core Research Facilities for Basic Science, Research Center for Medical Science, Jikei University School of Medicine, 3-25-8 Nishi-Shimbashi, Minato-ku, Tokyo 105-8461, Japan; E-Mail: atsubo@jikei.ac.jp

**Keywords:** chronic hepatitis C (CHC), hepatitis C virus (HCV), lipoprotein, triglyceride (TG), very-low-density lipoprotein (VLDL), VLDL sub-fractions

## Abstract

Reduced low-density lipoprotein (LDL) cholesterol level is a characteristic feature of dyslipidemia in chronic hepatitis C virus (HCV) infection. However, abnormality in serum triglyceride (TG) has not been fully investigated. To clarify the impact of HCV genotype 1b (G1b) infection and advanced fibrosis on serum TG profiles, TG concentrations in lipoprotein fractions were examined in fasting sera from 185 subjects with active or cleared HCV infection by high-performance liquid chromatography. Serum lipoproteins were fractionated into four classes: chylomicron, very low-density lipoprotein (VLDL), LDL, and high-density lipoprotein (HDL). Then, the significance of HCV G1b infection on TG levels in each lipoprotein fraction was determined using multiple regression models. We found that active HCV G1b infection was positively associated with high HDL-TG levels and low VLDL-TG levels, independent of other factors included in the regression model. In VLDL sub-fractions, active HCV infection was only found to be associated with low levels of large VLDL-TG. Similarly, advanced liver fibrosis in chronic HCV G1b infection was associated with high levels of LDL-TG, HDL-TG, and small VLDL-TG, independent of other clinical factors. These findings indicate that active HCV G1b infection and advanced fibrosis are closely associated with abnormal serum TG profiles.

## 1. Introduction

Chronic hepatitis C virus (HCV) infection is a major health problem worldwide and a leading cause of liver cirrhosis and hepatic carcinogenesis. Alteration of lipid metabolism in HCV infection has been investigated, and it is clear that HCV infection predisposes an individual not only to dyslipidemia, but also to hepatic steatosis [1] and advanced fibrosis. Moreover, dysregulated lipid metabolism in chronic HCV infection may predict the efficacy of interferon (IFN)-based antiviral therapy [2,3]. Although many studies have reported abnormal serum lipid levels in HCV infection, especially low levels of total cholesterol (TC) [4] and low-density lipoprotein cholesterol (LDL-C) [5], little is known about the serum triglyceride (TG) profile in HCV infection.

The liver, as a central organ in lipid metabolism, is involved in assembly and secretion of TG-rich very-low-density lipoprotein (VLDL) into circulation. In circulation, VLDL is catabolized by lipoprotein lipase (LPL) to a VLDL remnant called intermediate density lipoprotein (IDL), which is further metabolized to LDL by hepatic triglyceride lipase (HTGL) or incorporated into hepatocytes. Lipoprotein particles consist of free and esterified cholesterol, TGs, phospholipids, and apolipoproteins. Lipoproteins are classified into four major groups based on physicochemical characteristics: chylomicron (CM), VLDL, LDL, and high-density lipoprotein (HDL) [6].

Infectious HCV associates with lipoproteins to form lipo-viral particles in the blood. This association allows for particle entry into hepatocytes [7]. Both VLDLs and HCV lipo-viral particles are synthesized, assembled, and secreted from hepatocytes using similar metabolic pathways [8]. Therefore, VLDL is thought to be an indicator of dysregulated lipid metabolism in chronic HCV infection. Some studies have reported that the HCV core protein impairs VLDL assembly and secretion by suppressing the activity of microsomal triglyceride transfer protein (MTP) *in vitro* [9,10]. Among HCV genotypes, genotype 3 (G3) has been shown to be tightly associated with altered lipid metabolism [11]. However, as HCV G3 is very rare in Japan, we were not able to examine the disturbance of lipid profiles in patients with HCV G3.

Thus, in this study, we examined serum lipid profiles in Japanese patients with HCV G1b, which is the most prevalent HCV genotype in Japan [12,13]. Then, we describe the serum TG profiles of patients with active HCV G1b infection and further clarify the impact of HCV G1b infection on VLDL sub-fractions. In addition, we attempted to clarify whether there is a correlation between serum TG profile and liver fibrosis grade in HCV G1b infection.

## 2. Results

### 2.1. Features of Patients with Chronic Active HCV G1b Infection (Active HCV Group) and Those with Cleared HCV Infection Due to Anti-Viral Therapy (Sustained Virological Response Group; SVR Group)

Clinical features of active HCV and sustained virological response (SVR) groups are summarized in Table 1. The median age in the active HCV group was significantly higher than that in the SVR group, and the difference in gender between groups was marginal. There were no differences in body mass indices (BMIs) or hemoglobin A1c (HbA1c) levels. In routine laboratory tests, aspartate 2-oxoglutarate aminotransferase (AST), alanine 2-oxoglutarate aminotransferase (ALT), and γ-glutamyl transpeptidase (γ-GTP) levels were significantly higher in the active HCV group than in the SVR group, whereas albumin and platelet count were significantly lower. The difference in total bilirubin was marginal. As for serum lipids, TC and LDL-C levels were significantly lower in the active HCV group than in the SVR group, while TG and HDL-C levels were similar between the two groups.

**Table 1:** Comparison of clinical features between patients with chronic active HCV G1b infection (active HCV) and cleared HCV infection (SVR).

Discrete Traits	Active HCV (*n* = 103); *n* (%)		SVR (*n* = 82); *n* (%)				*p* Value	
Sex							0.08	
Male	36 (35)		40 (49)					
Female	67 (65)		42 (51)					
**Quantitative Traits**	**Median**	**(Q1–Q3)**	**Median**		**(Q1–Q3)**		***p* Value**	
Age (years)	71.0	63.0–78.0	64.0		56.0–72.8		0.001	
BMI (kg/m^2^)	21.9	20.1–24.2	22.6		20.9–24.5		0.1	
HbA1c (%)	5.5	5.2–5.8	5.6		5.3–6.0		0.1	
AST (IU/L)	41.0	29.5–63.5	22.0		19.0–28.0		<0.001	
ALT (IU/L)	33.0	23.0–53.5	16.0		13.0–22.8		<0.001	
Total bilirubin (mg/dL)	0.7	0.6–0.9	0.6		0.5–0.9		0.050	
γ-GTP (IU/L)	30.0	20.5–49.0	20.0		15.0–30.0		<0.001	
Albumin (g/dL)	4.1	3.8–4.3	4.4		4.2–4.6		<0.001	
Platelet (10^4^/μL)	15.0	11.2–20.4	18.0		14.8–21.8		0.002	
**Lipid Profiles**									
Total cholesterol (mg/dL)	174.0	151.5–192.0	196.0		176.3–217.0		<0.001	
Triglyceride (mg/dL)	88.0	67.0–112.0	89.5		70.3–135.0		0.4	
LDL cholesterol (mg/dL)	90.0	74.0–111.0	112.0		93.0–132.0		<0.001	
HDL cholesterol (mg/dL)	61.2	48.0–72.4	63.0		53.3–77.9		0.2	

### 2.2. Difference in TG Profiles in Serum Lipoprotein Fractions between Active HCV and SVR Groups

**Figure 1:**
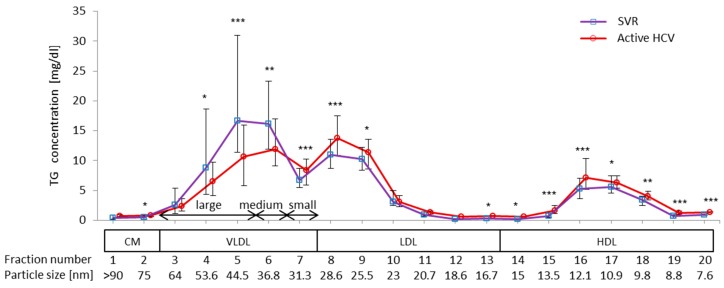
Comparison of triglyceride (TG) concentrations in lipoprotein sub-fractions between active HCV and SVR groups. Serum lipoprotein was fractionated by HPLC into 20 fractions according to the particle size, and the concentration of TG in each fraction was measured using an online detection system (Skylight Biotech, Inc., Akita, Japan). The fraction number and the mean particle size are shown at the bottom of the image (* *p* < 0.05; ** *p* < 0.01; *** *p* < 0.001).

The difference in TG concentrations for 20 serum lipoprotein fractions between active HCV and SVR groups is shown in Figure 1. The TG profile for serum lipoproteins was quite different between active HCV and SVR groups. TG concentrations in fractions 4–6 were significantly lower in the active HCV group than in the SVR group, but were higher in fractions 7–9 and fractions 13–20. There were no differences in TG profiles that were related to the HCV genotype of the past infection.

### 2.3. Impact of Chronic Active HCV G1b Infection on the Concentration of TG in the Four Major Classes of Lipoproteins

**Figure 2:**
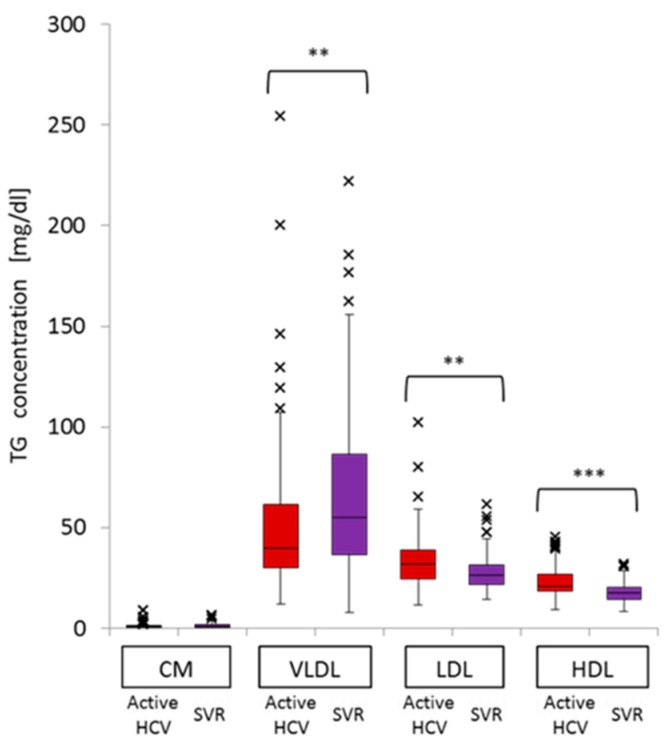
Comparison of TG concentrations in the four major classes of serum lipoproteins between active HCV and SVR groups. (** *p* < 0.01; *** *p* < 0.001).

TG concentrations in LDL-TG (median 31.7, interquartile range (IQR) 14.4 *vs.* median 26.4, IQR 9.8, *p* = 0.002) and HDL-TG (median 20.9, IQR 8.3 *vs.* median 17.6, IQR 6.1, *p* < 0.001) were significantly higher in patients with chronic active HCV infection than in the SVR group. In contrast, VLDL-TG (median 39.9, IQR 31.2 *vs.* median 55.1, IQR 49.9, *p* = 0.007) was significantly lower in patients with chronic active HCV infection than in the SVR group (Figure 2). The concentration of CM-TG was very low compared with those of the three other fractions.

After evaluating the significance of HCV G1b infection on TG concentrations in the four major classes of serum lipoproteins by multiple regression analysis, chronic active HCV G1b infection was found to be a significant factor independently associated with high serum levels of HDL-TG and low serum levels of VLDL-TG. Other factors that correlated with the serum level of VLDL-TG included gender and γ-GTP. Gender was also correlated with the serum level of HDL-TG. HCV G1b infection was not a factor associated with LDL-TG serum level. LDL-TG level was significantly affected by age, ALT, and γ-GTP (Table 2).

**Table 2:** Significance of chronic active HCV infection on serum levels of lipoproteins analyzed by multiple regression models.

	VLDL-TG			LDL-TG			HDL-TG		
	B	SE	*p* Value	B	SE	*p* Value	B	SE	*p* Value
Constant	2.4	44.4	1.0	9.2	16.2	0.6	22.7	8.6	0.01
HCV infection (Active HCV *vs.* SVR)	−13.8	6.9	0.047	1.6	2.5	0.52	3.1	1.3	0.02
Age	−0.01	0.2	0.96	0.2	0.08	0.01	0.005	0.04	0.9
Female gender	−12.7	5.8	0.03	−0.3	2.1	0.9	2.6	1.1	0.02
BMI	0.99	0.8	0.2	0.4	0.3	0.2	0.08	0.2	0.6
ALT	−0.02	0.1	0.8	0.1	0.04	0.03	−0.01	0.02	0.7
γ-GTP	0.2	0.08	0.01	0.06	0.03	0.04	0.03	0.01	0.09
Albumin	10.6	7.6	0.2	−2.7	2.8	0.3	−1.1	1.5	0.4
HbA1c	0.06	4.9	0.99	1.2	1.8	0.5	−0.6	1.0	0.5
Platelet	−0.3	0.2	0.3	−0.08	0.09	0.4	−0.01	0.05	0.8

### 2.4. Impact of Active HCV G1b Infection on TG Concentrations in the Three Sub-Fractions of Very Low-Density Lipoprotein (VLDL)

**Figure 3:**
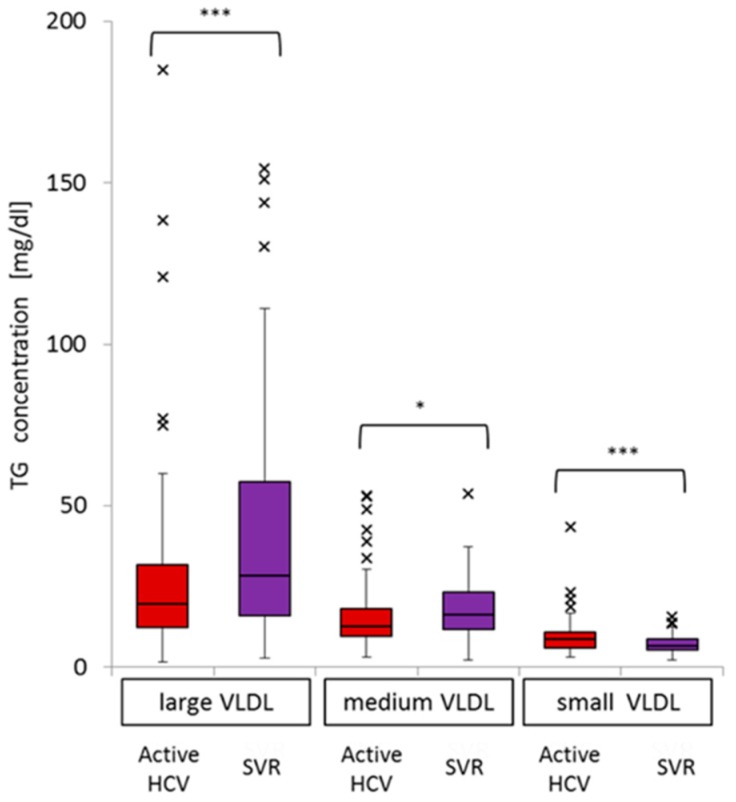
Comparison of the distribution of TG levels in the three VLDL sub-fractions for active HCV and SVR groups (* *p* < 0.05; *** *p* < 0.001).

In a further analysis of the distribution of TG in the three VLDL sub-fractions, large VLDL-TG (median 19.7, IQR 19.5 *vs.* median 28.3, IQR 41.2, *p* < 0.001) and medium VLDL-TG (median 12.5, IQR 8.5 *vs.* median 16.2, IQR 11.4, *p* = 0.01) levels were significantly lower in the active HCV group than in the SVR group. By contrast, small VLDL-TG levels (median 8.68, IQR 4.8 *vs.* median 6.73, IQR 3.22, *p* < 0.001) were significantly higher in the patients with active HCV than in the SVR group (Figure 3).

Using multiple regression analysis, we found that chronic active HCV G1b infection was significantly associated with a lower level of large VLDL-TG and marginally associated with lower levels of medium VLDL-TG. Chronic active HCV G1b infection was not associated with small VLDL-TG levels. Other factors associated with large VLDL-TG levels included gender, γ-GTP, and albumin; medium VLDL-TG levels were associated with γ-GTP; and small VLDL-TG levels were associated with albumin (Table 3).

**Table 3:** Significance of active chronic HCV infection in the distribution of TG in the three VLDL sub-fractions analyzed by multiple regression models.

	Large VLDL-TG			Medium VLDL-TG			Small VLDL-TG		
	B	SE	*p* Value	B	SE	*p* Value	B	SE	*p* Value
Constant	−28.2	33.2	0.4	12.1	11.7	0.3	18.5	5.6	0.001
HCV infection (Active HCV *vs*. SVR)	−10.6	5.1	0.04	−3.2	1.8	0.08	−0.04	0.9	0.97
Age	−0.06	0.2	0.7	0.003	0.06	0.96	0.04	0.03	0.1
Female gender	−10.2	4.3	0.02	−2.9	1.5	0.06	0.3	0.7	0.7
BMI	0.7	0.6	0.2	0.2	0.2	0.4	0.1	0.1	0.3
ALT	−0.04	0.07	0.6	−0.002	0.03	0.93	0.02	0.01	0.1
γ-GTP	0.1	0.06	0.03	0.05	0.02	0.02	0.02	0.01	0.049
Albumin	14.3	5.7	0.01	−0.3	2.00	0.9	−3.4	0.9	<0.001
HbA1c	−0.4	3.7	0.9	0.8	1.3	0.5	−0.3	0.6	0.6
Platelet	−0.2	0.2	0.4	−0.09	0.06	0.2	−0.02	0.03	0.5

### 2.5. Significance of Advanced Liver Fibrosis on Serum Levels of VLDL-TG, LDL-TG, and High-Density Lipoprotein (HDL)-TG in Patients with Chronic Active HCV G1b Infection

In 51 patients with advanced liver fibrosis and a FIB-4 index score above 3.25, the median age, as well as ALT and AST levels, were higher, and serum albumin level and platelet counts were lower, than those of 52 patients with mild-moderate fibrosis. There were fewer patients of male gender and non-TT IFNL3 genotype in the advanced fibrosis group than in the mild-moderate fibrosis group, but the differences were marginal. HCV-RNA and HbA1c levels were similar between the two groups. As for serum lipids, TC and LDL-C were significantly lower in the advanced fibrosis group, while TG and HDL-C serum levels were similar between the two groups (Table 4).

**Table 4:** Comparison of clinical features and the interferon lambda (IFNL) 3 genotype between HCV G1b patients with advanced fibrosis and those with mild-moderate fibrosis.

Discrete Traits	Advanced Fibrosis (*n* = 51); *n* (%)		Mild-Moderate Fibrosis (*n* = 52); *n* (%)		*p* Value
Sex					0.07
Male	13 (25)		23 (44)		
Female	38 (75)		29 (56)		
IFNL3 genotype (rs8099917)					0.06
TT	40 (78)		33 (63)		
Non-TT	9 (18)		18 (35)		
Not done	2 (4)		1 (2)		
**Quantitative Traits**	**Median**	**(Q1–Q3)**	**Median**	**(Q1–Q3)**	***p* Value**
Age (years)	75.0	68.5–80.5	66.0	52.8–73.0	<0.001
BMI (kg/m^2^)	22.1	20.1–24.1	21.5	20.3–24.4	0.97
HbA1c (%)	5.5	5.0–5.8	5.5	5.3–5.8	0.4
HCV-RNA (log IU/mL)	6.5	6.0–6.8	6.7	5.8–7.1	0.2
AST (IU/L)	58.0	38.5–82.0	30.5	25.0–42.0	<0.001
ALT (IU/L)	44.0	27.0–62.5	30.0	19.8–47.3	0.01
Total bilirubin (mg/dL)	0.7	0.6–0.9	0.8	0.6–0.8	0.7
γ-GTP (IU/L)	33.0	22.0–48.5	30.0	18.0–49.5	0.5
Albumin (g/dL)	4.0	3.6–4.3	4.2	4.0–4.3	0.01
Platelet (10^4^/μL)	11.3	8.5–14.3	19.6	15.6–23.0	<0.001
**Lipid Profiles**					
Total cholesterol (mg/dL)	168.0	142.0–187.5	176.5	160.8–193.3	0.04
Triglyceride (mg/dL)	89.0	68.5–113.5	86.5	63.0–105.3	0.6
LDL cholesterol (mg/dL)	84.0	69.0–99.5	96.0	79.8–112.5	0.04
HDL cholesterol (mg/dL)	58.4	46.9–68.0	63.1	50.3–73.2	0.2

### 2.6. Difference in VLDL-TG, LDL-TG, and HDL-TG Serum Levels between Patients with Advanced Liver Fibrosis and Those with Mild-Moderate Fibrosis

**Figure 4:**
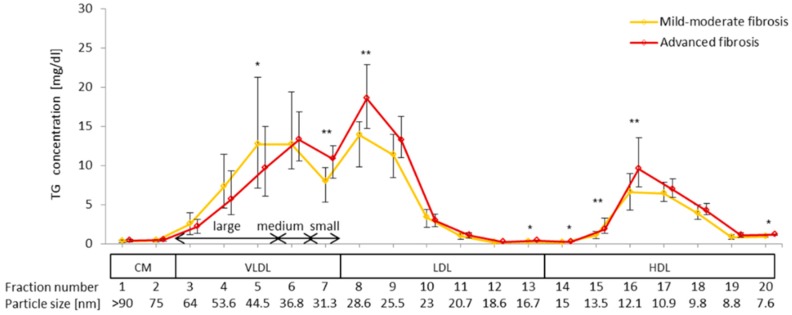
Comparison of TG concentrations in lipoprotein sub-fractions between patients with advanced fibrosis and those with mild-moderate fibrosis. Serum lipoprotein was fractionated by HPLC into 20 fractions according to the particle size, and the concentration of TG in each fraction was measured using an online detection system (Skylight Biotech, Inc., Akita, Japan). The fraction number and the mean particle size of each fraction are presented at the bottom of the image (* *p* < 0.05; ** *p* < 0.01).

Serum TG concentrations in 20 lipoprotein fractions of HCV G1b-infected patients with advanced and mild-moderate fibrosis were measured (Figure 4). The concentrations of TG in fractions 5 and 14 were significantly lower in patients with advanced fibrosis than in those with mild-moderate fibrosis, while those of fractions 7, 8, 13, 15–16, and 20 were significantly higher in patients with advanced fibrosis.

### 2.7. Impact of Advanced Liver Fibrosis on TG Concentration in the Four Serum Lipoprotein Classes in Patients with Chronic Active HCV G1b Infection

**Figure 5:**
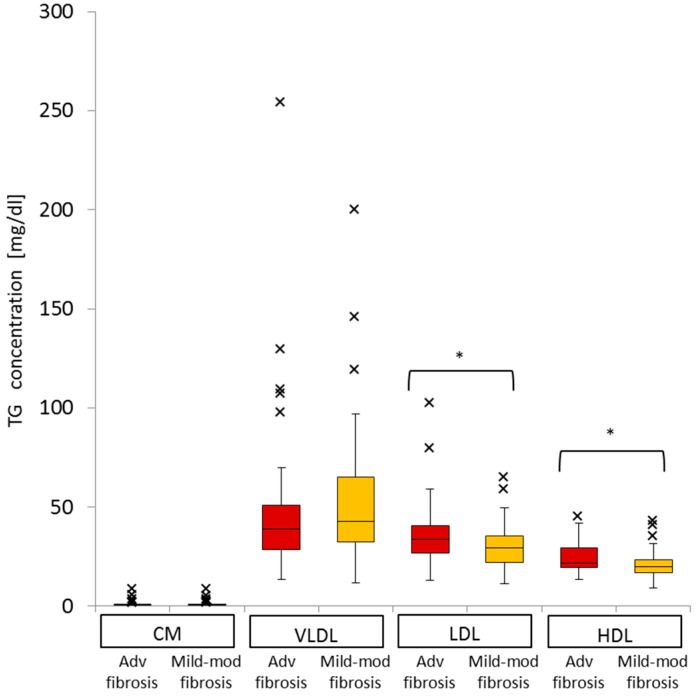
Comparison of the distribution of TG in the four major classes of serum lipoproteins between patients with advanced fibrosis and those with mild-moderate fibrosis (Adv, advanced; Mod, Moderate; * *p* < 0.05).

With regard to the distribution of TG in the four major classes of serum lipoprotein, LDL-TG (median 33.7, IQR 13.8 *vs.* median 29.2, IQR 13.5, *p* = 0.02) and HDL-TG (median 21.7, IQR 10.1 *vs.* median 20.0, IQR 6.6, *p* = 0.03) were significantly higher in patients with advanced fibrosis, while VLDL-TG (median 38.7, IQR 22.3 *vs.* median 42.8, IQR 32.9, *p* = 0.3) was not different between the advanced and mild-moderate fibrosis groups (Figure 5). The concentration of CM-TG was very low compared with those of the three other fractions.

We found advanced fibrosis to be a significant factor independently associated with higher levels of serum LDL-TG and HDL-TG, but not VLDL-TG. Along with advanced fibrosis, the level of serum LDL-TG was associated with the quantity of HCV-RNA, and the serum level of HDL-TG was associated with the INFL3 genotype. Serum VLDL-TG level was associated with HCV-RNA (Table 5).

**Table 5:** Significance of advanced liver fibrosis in the concentrations of VLDL-TG, LDL-TG, and HDL-TG, as analyzed using multiple regression models.

	VLDL-TG			LDL-TG			HDL-TG		
	B	SE	*p* Value	B	SE	*p* Value	B	SE	*p* Value
Constant	10.8	42.6	0.8	−23.9	21.4	0.3	15.4	10.8	0.2
Advanced fibrosis	−2.8	6.5	0.7	10.4	3.3	0.002	4.2	1.7	0.01
IFNL3 (Non-TT)	2.9	7.2	0.7	−2.1	3.6	0.6	4.3	1.8	0.02
Female gender	−13.1	6.7	0.055	−4.0	3.4	0.2	0.2	1.7	0.9
HCV-RNA	7.7	3.8	0.04	4.8	1.9	0.01	0.8	1.0	0.4
BMI	0.6	1.0	0.6	0.4	0.5	0.4	0.2	0.2	0.4
HbA1c	−2.8	5.0	0.6	2.9	2.5	0.25	−1.1	1.3	0.4

### 2.8. Significance of Advanced Fibrosis for Serum TG Concentrations in the Three Subclasses of VLDL

**Figure 6:**
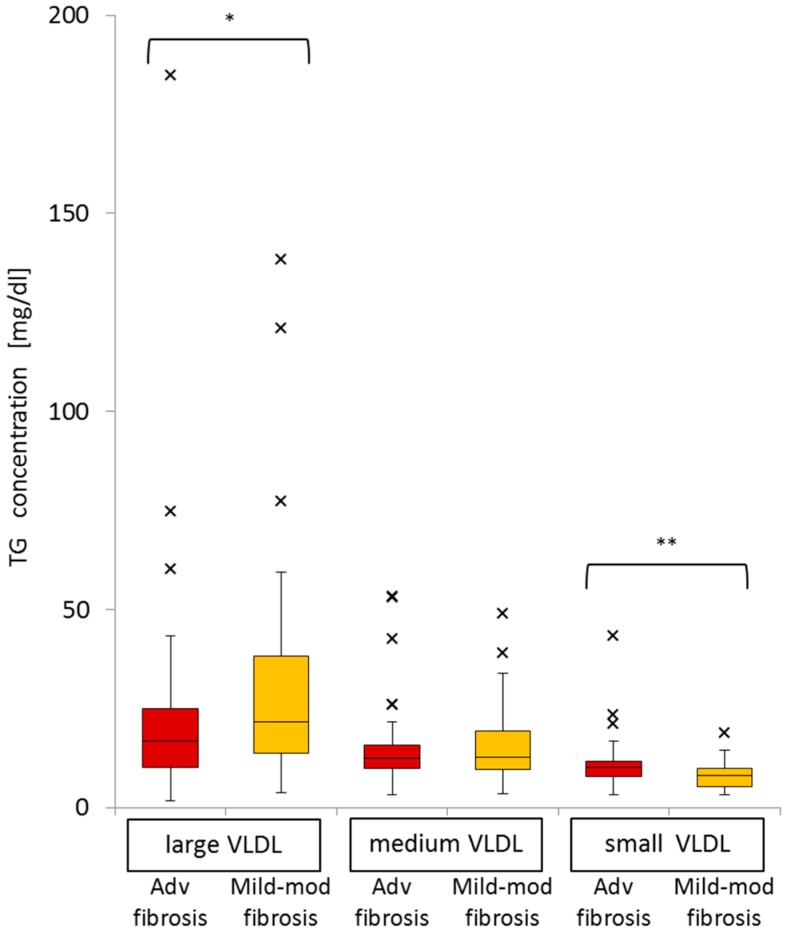
Comparison of the distribution of TG in VLDL sub-fractions between patients with advanced fibrosis and those with mild-moderate fibrosis (Adv; advanced, Mod; moderate, * *p* < 0.05; ** *p* < 0.01).

In VLDL sub-fractions, small VLDL-TG levels (median 10.0, IQR 3.82 *vs.* median 7.98, IQR 4.40, *p* = 0.002) were significantly higher in advanced fibrosis patients than in mild-moderate fibrosis patients, but large VLDL-TG levels (median 16.8, IQR 14.6 *vs.* median 21.5, IQR 24.4, *p* = 0.03) were significantly lower. Medium VLDL-TG levels (median 12.4, IQR 5.90 *vs.* median 12.7, IQR 9.83, *p* = 0.6) were not different between the advanced and mild-moderate fibrosis groups (Figure 6).

We found that advanced fibrosis was significantly associated with higher levels of small VLDL-TG, but not with large and medium VLDL-TG. The serum level of large VLDL-TG was related to gender. Other factors, including the IFNL3 genotype and the quantity of HCV-RNA, had no association with TG levels in the three subclasses of VLDL (Table 6).

**Table 6:** Impact of advanced fibrosis on the distribution of TG in VLDL sub-fractions analyzed using multiple regression models.

	Large VLDL-TG			Medium VLDL-TG			Small VLDL-TG		
	B	SE	*p* Value	B	SE	*p* Value	B	SE	*p* Value
Constant	7.3	27.9	0.8	0.0	14.2	1.0	3.5	8.0	0.7
Advanced fibrosis	−6.1	4.3	0.16	0.1	2.2	0.98	3.2	1.2	0.01
IFNL3 (Non-TT)	6.0	4.7	0.2	−1.3	2.4	0.6	−1.7	1.4	0.2
Female gender	−9.2	4.4	0.04	−3.6	2.2	0.1	−0.3	1.3	0.8
HCV-RNA	4.3	2.5	0.1	2.3	1.3	0.1	1.2	0.7	0.1
BMI	0.4	0.6	0.5	0.08	0.3	0.8	0.04	0.2	0.8
HbA1c	−2.4	3.3	0.5	0.2	1.7	0.91	−0.6	0.9	0.5

## 3. Discussion

In the present study, we examined the serum TG profiles of patients with chronic active HCV G1b infection and of those with cleared HCV infection by using their fasting serum samples, which were fractionated by HPLC. The detailed serum TG profiles in chronic HCV infection have never been fully investigated. Using multiple regression analysis, we found that HCV infection is associated with low levels of VLDL-TG and high levels of HDL-TG, independent of other clinical factors. Moreover, HCV infection is independently associated with a low level of large VLDL-TG in sub-fractions of VLDL.

The lack of healthy donors as non-HCV infected controls may be one of the limitations of the present study. However, the composition of TG in lipoprotein fractions in our SVR group is similar to those in normal Japanese adults [14]. The mean weight ratio of cholesterol to TG (C/T ratio) in our SVR group and Japanese normal adults is as follows: C/T ratio in LDL, 4.08 and 3.69; C/T ratio in HDL, 3.09 and 3.23, respectively. The C/T ratio of the active HCV group was fairly low (2.67 in LDL, 2.19 in HDL). This finding indicates that the composition of TG in lipoprotein fractions tends to normalize after eradication of HCV. Therefore, it is possible to compare the lipid profiles of SVR groups and active HCV patients in order to elucidate the influence of HCV infection on lipoprotein metabolism.

There are considerable differences in the clinical backgrounds between the active HCV and SVR groups. Insulin resistance syndrome (metabolic syndrome), including dysmetabolic factors such as BMI and HbA1c, could contribute to the development of resistance to IFN-based antiviral therapy in chronic HCV patients [15,16]. In the present study, we did not perform a homeostasis model assessment as an indicator of insulin resistance. However, there were no differences in BMI or HbA1c between the SVR and active HCV groups. Moreover, when these metabolic factors were included as variables in multiple regression analysis, HCV infection emerged as an independent factor associated with the distribution of TG in our cohort.

In our study, total TG concentration was similar between the active HCV and SVR groups. There is no consensus on TG levels in chronic HCV infection. Some studies have reported that TG levels were similar between patients with active HCV and control groups [17,18,19], while others reported a decreased level of TG during active HCV infection [20,21]. Significant differences were noted in the distribution of TG in lipoprotein fractions. A decrease in the serum VLDL-TG/non-VLDL-TG ratio was reported to be a feature of early stage of chronic hepatitis C (CHC) [22]. However, the detailed distribution of non-VLDL-TG has not been examined. Our results clearly indicate that an increase in HDL-TG is associated with HCV infection. Therefore, the increase in HDL-TG in HCV infection could account for the observed decrease in the VLDL-TG/non-VLDL-TG ratio.

The increased HDL-TG levels associated with HCV infection strongly suggest that excessive TG in apolipoprotein B-100 (apoB-100)-related lipoproteins (VLDL and LDL) is transported to HDL, because TG in HDL is chiefly derived from transportation of excessive TG in apoB-100-related lipoproteins by the function of cholesterol ester transfer protein (CETP) [23]. We recently discovered that active HCV infection is associated with an increase in CETP levels (data submitted). The decreased VLDL-TG levels associated with HCV infection were consistent with the findings of previous reports [9,10] that suggest that VLDL assembly and secretion are suppressed in active HCV infection. However, this finding seems to contradict the finding that there is excessive TG in apoB-100-related lipoproteins in HCV infection. This contradiction could be most reasonably explained through incomplete hydrolysis of VLDL in HCV infection, which would lead to abnormally high TG content in LDL. Serum levels of apoB-100 and LDL-C have been consistently reported to be decreased with HCV infection [4,21,24,25], strongly suggesting that there is a decrease in LDL particles in HCV infection. Therefore, even though LDL-TG is not increased in HCV infection, TG is excessive in LDL particles, which is supported by the abnormally low C/T ratio of LDL in HCV infection.

Our study on sub-fractionated VLDL-TG indicated that large VLDL-TG is reduced in HCV infection. The content of TG in VLDL secreted from the liver is variable depending on the situation [26]. Expression of HCV core protein impairs the synthesis of VLDL [10], and causes a decrease in VLDL particle size in hepatocytes from transgenic mice [9]. Our finding that large VLDL-TG levels are low in the active HCV group is consistent with the particle size of VLDL becoming smaller with increased expression of HCV core protein.

Advanced fibrosis with chronic HCV G1b infection was found to be independently associated with high levels of LDL-TG, HDL-TG, and small VLDL-TG by multiple regression models. However, advanced fibrosis is not an independent factor associated with a decrease in VLDL-TG. High levels of small VLDL-TG and LDL-TG in advanced fibrosis may be the result of reduced hydrolytic activity, particularly a reduction in HTGL activity [27] because TG in small VLDL is mainly hydrolyzed by HTGL. Reduced HTGL activity impairs hydrolysis of VLDL-TG and causes an increase in the serum level of small VLDL-TG and an increase in incompletely hydrolyzed LDL, which has abundant TG. Thus, abundant TG in LDL is transferred to HDL by the same mechanisms described earlier. As an additional finding, we noticed that the minor IFNL3 genotype, which was reported to affect not only the efficacy of anti-HCV therapy but also lipid metabolism [28], was an independent factor associated with increased TG concentration in HDL.

From our detailed study on serum abnormal TG distribution, the pathogenesis of progressive atherosclerosis, which is one of the systemic complications of chronic HCV infection [29,30,31], could be partly explained. Many previous studies reported that several direct and indirect mechanisms involving liver steatosis [32] and fibrosis [33], type 2 diabetes and insulin resistance [34], secretion of inflammatory cytokines [35], oxidative stress [36], endotoxemia [37], cryoglobulinemia [38], and other factors might contribute to the progression of atherosclerosis in HCV infection. However, the significance of disturbed lipoprotein metabolism in chronic HCV infection with respect to the progression of atherosclerosis has not been extensively studied. In the present study, we found that an increase of LDL-TG and small VLDL-TG (relevant to IDL or VLDL remnant) is an independent factor associated with chronic active HCV G1b infection with advanced fibrosis. In contrast, BMI and HbA1c levels were not associated with abnormal TG distribution. As VLDL remnants have been unequivocally shown to be atherogenic [39,40], a high level of small VLDL-TG may be one of the risk factors of progressive atherosclerosis in HCV infection with advanced fibrosis. In a very recent study, advanced fibrosis with HCV infection was reported to promote atherosclerosis independently of common cardiovascular risk factors [41]. Therefore, the abnormal TG metabolism in chronic active HCV G1b infection with advanced fibrosis may be one of the causes of progressive atherosclerosis independent of other risk factors. However, as we did not examine the degree of atherosclerosis in our patients, further studies are required for establishing the significance of disturbed TG metabolism in the progression of atherosclerosis.

## 4. Experimental Section

### 4.1. Patients

In total, 185 Japanese patients with active or cleared chronic HCV infection undergoing care at Jikei University Katsushika Medical Center from June 2013 to December 2014 were enrolled randomly in this study. Patients who had hepatocellular carcinoma, decompensated cirrhosis, other types of hepatitis, co-infected with human immunodeficiency virus or other viruses, habitual alcohol abuse, treatment of dyslipidemia, or diabetes were excluded from this study. Patients who were under antiviral therapy or finished antiviral therapy within 6 months of the time of enrollment were also excluded.

Among the enrolled patients, 103 patients had chronic active HCV G1b infection and 82 patients had undergone SVR defined as HCV-RNA being undetectable at least 24 weeks after receiving IFN-based antiviral therapy [42]. Almost all patients with cleared HCV infection were genotyped before receiving antiviral therapy, and 48 patients had been previously infected with G1b, while 33 had been infected with G2. The genotype of one patient was unknown.

### 4.2. Study Design

The difference in TG profiles in the serum lipoprotein subclasses between patients with chronic active HCV G1b infection and cleared HCV infection were determined. Peripheral blood was obtained from all participants after an overnight fast of at least 10 h. The freshly separated serum was fractionated according to lipoprotein size, and the concentration of TG in each fraction was measured. The correlation of chronic active HCV G1b infection with serum TG profiles was examined.

In addition, liver fibrosis grade in chronic active HCV G1b infection was assessed, and the association of advanced liver fibrosis with serum TG profiles was investigated. The protocol for this trans-sectional study complied with the 2004 standards of the Declaration of Helsinki and current ethical guidelines and was approved by the human ethics review committee of the Jikei University School of Medicine (21-032 5610, 1 February 2010, a study of the association between lipoprotein metabolism and pathology in patients with CHC). Written informed consent was obtained from all patients who enrolled in this study.

### 4.3. Analysis of Serum TG Profiles in Lipoprotein Subclasses

Serum lipoproteins were fractionated by HPLC into 20 fractions and the concentration of TG in each fraction was measured using an online detection system (Skylight Biotech, Inc., Akita, Japan). According to the manufacturer’s instructions, serum lipoproteins were classified into four major fractions (CM, VLDL, LDL, and HDL) by particle size. Among 20 fractions, fractions 1–2 were classified as CM, fractions 3–7 as VLDL, fractions 8–13 as LDL, and fractions 14–20 as HDL. Furthermore, the VLDL fraction was divided into three sub-fractions: fractions 3–5 were large VLDL, fraction 6 was medium VLDL, and fraction 7 was small VLDL [43,44]. The mean particle size for each fraction is shown in Figure 1.

### 4.4. Demographic and Basic Laboratory Tests

Demographic data including age, gender, and BMI, as well as blood samples for analyzing serum TG profiles including AST, ALT, γ-GTP, albumin, total bilirubin, platelet count, and HbA1c were collected from each patient.

### 4.5. Evaluation of Serum Lipid Profiles

TC, TG, and HDL-C were directly assayed using a commercially available conventional kit (Kyowa Medex, Tokyo, Japan). The concentration of LDL-C was calculated using the Friedewald equation because there were no patients whose serum fasting TG levels were ≥400 mg/dL [45].

### 4.6. Measurement of Serum HCV-RNA Level and Genotyping of Interferon Lambda (IFNL) 3

The level of serum HCV-RNA was quantified with the COBAS TaqMan HCV test (Roche Diagnostics Japan, Tokyo, Japan). The rs8099917 single nucleotide polymorphism (SNP) near IFNL3 gene was determined by real-time polymerase chain reaction (PCR), using the TaqMan SNP genotyping assay and the 7500 fast real-time PCR system (Applied Biosystems, Foster City, CA, USA). The genotype of IFNL3 (rs8099917) was classified as TT or non-TT (TG or GG) [46].

### 4.7. Evaluation of Liver Fibrosis in Patients with Chronic Active HCV G1b Infection

The grade of hepatic fibrosis in chronic active HCV infection was evaluated by using the FIB-4 index, a simple and reliable noninvasive liver fibrosis marker that combines standard biochemical values (platelet count, ALT, and AST) and age. The FIB-4 index was calculated using the following equation: (age × AST)/(platelet count (×10^9^ L^−1^) × ALT^0.5^), and a score above 3.25 was classified as advanced fibrosis [47,48].

### 4.8. Statistics

Continuous data were expressed as median (Q1–Q3) or median (interquartile range; IQR). Categorical data were expressed as numbers (%). Comparisons between two groups were performed by using the χ^2^ test or Mann-Whitney U test.

To determine the significance of the association of chronic active HCV infection with TG concentration in each lipoprotein fraction, multiple regression models were generated. Each lipoprotein subclass (VLDL, LDL, or HDL) or each sub-fraction of VLDL (large VLDL, medium VLDL, or small VLDL) was selected as a dependent variable, and nine variables were selected as independent variables, including three variables related to demographic data (age, gender, and BMI), four variables related to laboratory data (ALT, γ-GTP, albumin, platelet count, and HbA1c), and one variable related to the status of HCV infection (active or cleared from HCV infection). Categorical data was coded as 0 or 1, with female gender as 1 and male gender as 0, and active HCV infection as 1 and cleared HCV infection (SVR) as 0.

To verify the impact of advanced liver fibrosis on serum TG profiles in chronic active HCV G1b infection, similar multiple regression models were constructed. Each lipoprotein subclass (VLDL, LDL, or HDL) or each sub-fraction of VLDL (large VLDL, medium VLDL, or small VLDL) was selected as a dependent variable, and gender, BMI, FIB-4 index, HCV-RNA, HbA1c, and IFNL3 genotype were selected as independent variables. Demographic and laboratory data included in the FIB-4 index (age, AST, ALT, and platelet count) were excluded from being considered as independent variables. Categorical data was coded as 0 or 1, with IFNL3 genotype non-TT as 1 and TT as 0, and FIB-4 index <3.25 as 1 and >3.25 as 0.

All statistical analyses were performed using STATISTICA software, version 6 (StatSoft Japan Inc. Tokyo, Japan). Two-tailed *p* < 0.05 was considered significant, whereas 0.05 < *p* < 0.1 was marginal. *p* values below 0.001 were expressed as *p* < 0.001. Multicollinearity of each multiple regression analysis was surveyed to verify the reliability of our analysis, and the variance inflation factor was below 5, which indicated that our models were statistically reliable.

## 5. Conclusions

Chronic active HCV G1b infection is associated with altered serum TG profiles, characterized by reduced levels of VLDL-TG and increased levels of HDL-TG. In VLDL sub-fractions, a decrease in the level of large VLDL-TG is associated with HCV infection. Meanwhile, advanced fibrosis among patients with chronic active HCV G1b infection was associated with an increase in LDL-TG, HDL-TG, and small VLDL-TG. These findings may indicate that the secretion of large VLDL is impaired in HCV infection and that the catabolism of small VLDL is diminished in advanced liver fibrosis, which is caused by chronic active HCV infection. An increase in small VLDL-TG may be one of the causes of progressive atherosclerosis complicated with chronic active HCV infection with advanced fibrosis.
